# Subchronic Study of a White Kidney Bean (*Phaseolus vulgaris*) Extract with *α*-Amylase Inhibitory Activity

**DOI:** 10.1155/2019/9272345

**Published:** 2019-11-22

**Authors:** Guangqiu Qin, Fang Wang, Huili Liang, Song Tang, Kamran Shekh, Yanwu Wang, Bin Li, Baiqing Dong, Pingjing Wen

**Affiliations:** ^1^Department of Toxicology, Guangxi University of Chinese Medicine, Nanning, China; ^2^Institute of Toxicology, Guangxi Center for Disease Prevention and Control, Nanning, China; ^3^Department of Environmental Toxicology, National Institute of Environmental Health, Chinese Center for Disease Control and Prevention, Beijing, China; ^4^Center for Global Health, School of Public Health, Nanjing Medical University, Nanjing, Jiangsu, China; ^5^Toxicology Centre, University of Saskatchewan, Saskatoon, Saskatchewan, Canada

## Abstract

Common bean extract as a dietary supplement has received increased attention globally owing to its *α*-amylase inhibitory activity. The objective of this study was to evaluate the toxicity of a white kidney bean (*Phaseolus vulgaris*) extract by a repeated-dose 90-day subchronic oral toxicity study in Sprague-Dawley rats. In the subchronic toxicity study, 80 rats were orally administrated with white kidney bean extract at doses of 4, 2, and 1 g/kg body weight daily for 90 days. The results showed that the white kidney bean extract at doses up to 4 g/kg/day did not induce significant changes in body weight, organ weight, food consumption, hematology, serum biochemistry, and histopathology in rats, as compared to the control. The no-observed-adverse-effect level (NOAEL) of white kidney bean extract was determined to be >4 g/kg/day for both male and female rats, under the experimental conditions of this study.

## 1. Introduction

Common bean (*Phaseolus vulgaris*) belongs to the family Fabaceae and genus *Phaseolus*. Common bean is rich in starch, dietary fiber, protein, unsaturated fatty acids, and vitamin [1, 2]. It is widely consumed throughout the world, accounting for 50% of grain legumes indirect human consumption [3]. It is recognized as the major source of dietary protein in many Asian, Latin American, and African countries [4, 5]. For example, in China, the consumption rates of beans in adults aged 18–59 years were 62.1%; the average amount of consumption in the whole population was 1.1 g/day, in 2015 [6]. In Tunisia, beans provided 0.50 g/capita/day proteins for Tunisian population in 2009 [7].

In addition to nutrients, common beans from different accessions have been reported to contain high level of alpha-amylase inhibitors (*α*-AI) [5]. Alpha-amylase (*α*-1,4-glucan-4-glucanohydrolase) is an important enzyme for many organisms. It catalyzes the hydrolysis of starch into oligosaccharides, which contributes to the energy supply of organisms. *α*-AI, also called “starch blockers” or “carbohydrates blockers,” are a class of substances showing inhibitory activity against *α*-amylase [8]. Attributed mainly to *α*-AI, extracts of common beans have been shown to have multiple specific functions such as antiobesity, antidiabetic, and glycaemic control activities [9–15]. For example, the acute oral intake of common bean extract (50 mg/kg bw) reduced the increase in glycaemia in adult Wistar rats treated with a starch load, without modifying the insulin response [11]. Moreover, common bean extract decreased glycaemia levels in rats in both acute and subchronic studies [16]. Due to their ability to inactivate amylase in the intestinal lumen, products containing common bean extracts have been marketed as dietary supplements for weight and glycaemic control [9].

Irrespective of their nutritional and health benefits, common beans also contain a variety of antinutritional substances such as trypsin inhibitor, lectins, polyphenols (condensed tannins and anthocyanins), and some oligosaccharides [2]. These antinutritional factors may affect the digestibility and bioavailability of nutrients and limit their consumption [17]. For example, trypsin inhibitors could interfere in protein digestion and decrease the bioavailability of sulfur-containing amino acids, causing metabolic disturbance [18]. Phytohemagglutinins, a class of lectins that are present at high levels in raw common beans, may affect animal growth by interfering with the digestion and absorption of nutrients in the gastrointestinal tract [18]. Consumption of common beans has been observed to induce negative effects including reduced feed efficiency, impaired weight gain, histopathological changes, and even death in animals [19]. In humans, consumption of raw or undercooked kidney beans may induce severe but transient gastrointestinal disturbances such as nausea, vomiting, diarrhea, and abdominal pain [20].

Although the common bean extract has the potential as a nutraceutical additive, relevant information on its systemic toxicity and safety evaluation is scarce. To date, only a few studies have been conducted to evaluate the toxicity of common bean extract from different sources [19–21]. Given the potential utility of *α*-AI-containing common beans extract as a dietary supplement, it is necessary to conduct a comprehensive toxicological assessment to demonstrate the safety of such a product for possible use in food. Therefore, the objective of this study was to investigate the toxicity of a white kidney bean (*P. vulgaris*, WKB) extract by a 90-day subchronic oral toxicity study in rats. This study is expected to provide useful information towards the safe and effective utilization of common bean extract in food.

## 2. Materials and Methods

### 2.1. Test Material

The test material used in the toxicity study was a dried aqueous extract of the common white kidney beans (WKBs, *P. vulgaris*). WKBs were purchased from a local market in Kunming, China, where they are widely planted and consumed. The beans were collected at one time from the same farm under the same cultivating conditions. Alpha-amylase inhibitors (*α*-AIs) were extracted from the beans as described previously, with a little modification [22]. In brief, beans were milled into a fine powder by sieving through a 60-mesh sieve. The powder was suspended in 1.5% NaCl solution (1 : 8, w : v) and stirred for 4 h under room temperature. The mixture was then centrifuged at 12,000*g* for 1 h. The supernatant was collected, heated at 70°C for 20 min at pH 5.3, and centrifuged at 12,000*g* for 20 min to remove heat-labile proteins. The supernatant was mixed with ethanol (3 : 7, v : v), cooled in refrigerator at 4°C for 1 h, and then centrifuged at 12,000*g* for 30 min. The precipitates were collected, freeze-dried, and used as the WKB extract in this study. The yield of the WKB extract was 2.83 g/100 g dried bean. The extract was stored at −20°C for further use.

The *α*-amylase inhibitory activity of the WKB extract was determined to be 2,157 U/g, according to the method described previously [23]. In brief, the WKB extract was dissolved in a buffer solution (15 mmol/L NaOH, 20 mmol/L CaCl_2_, and 0.5 mol/L NaCl, pH 5.6) containing 40 U/mL of porcine pancreatic *α*-amylase (Sigma-Aldrich, St. Louis, USA). The solution was incubated in a water bath at 37°C for 30 min. Afterwards, 400 *μ*L soluble starch solution (2%, w : v) was added to 200 *μ*L of the solution and incubated for 1 min. To stop the reaction, 800 *μ*L of 3,5-dinitrosalicylic acid (0.65%, w : v) was added and the mixture was incubated in boiling water for 10 min. The absorbance of the solution was measured at 520 nm using a Shimadzu UV-1700 UV-visible spectrophotometer (Shimadzu, Kyoto, Japan).

### 2.2. Polypeptide Pattern Analysis

The polypeptide pattern of the WKB extract was analyzed by SDS-PAGE [24] and compared to a commercial product Phase 2® WKB Extract (NOW Foods, Bloomingdale, USA). In brief, samples were dissolved in Tris-HCl buffer (1 mol/L, pH 6.8) and loaded onto the gel with 0.2 mg soluble protein. Molecular mass standards (10–180 kDa; Thermo Fisher, USA) were also loaded in a separate well on each gel. Electrophoresis was performed in 62.5 mmol/L Tris-HCl buffer with 3.4 mmol/L SDS for 2 h (Bio-Rad, USA). The electrophoresis results were visualized by Coomassie brilliant blue staining and analyzed with Tanon 5200 Digital Gel Image System (Tanon, China).

### 2.3. Animal Handling and Husbandry

Specific pathogen-free (SPF) Sprague-Dawley (SD) rats (3-week-old, 60–80 g) and conventional diets were purchased from the Medical Experimental Animal Center of Guangdong Province (Guangzhou, Guangdong, China). Animals were kept under 12 h light/dark cycle within the temperature range of 23 ± 1°C and 60 ± 5% relative humidity. The animals were housed in polycarbonate cages with free access to conventional diets and sterilized tap water. Animals were acclimated to laboratory conditions for a period of 3 days prior to the experiments. The protocols for animal studies were reviewed and approved by the Animal Experimentation Ethics Committee at Guangxi Center for Disease Prevention and Control (Nanning, Guangxi, China).

### 2.4. Subchronic Oral Toxicity Study in Rats

#### 2.4.1. Oral Administration

A subchronic 90-day oral toxicity study was conducted following a standard protocol set by the Ministry of Health of China [25–27]. A total of 80 healthy rats were randomly divided into 4 groups (3 treatment groups and 1 control group), with 10 males and 10 females in each group. Male and female rats were housed separately in polycarbonated cages with a maximum of 3 rats per cage. Rats in the treatment groups were given 4, 2, and 1 g/kg bw of WKB extract (dissolved in distilled water) by gavage for 90 days, respectively. Doses were chosen according to the recommendation of the standard protocol [25]. The maximum tolerated dose (MTD) for oral intake of WKB extract was determined to be greater than 16,000 mg/kg bw in mice in the 14 d acute oral toxicity study (data not shown). Therefore, the maximum dose 4 g/kg bw was selected as the high dose and 2 and 1 g/kg as the middle and small doses, using a common ratio of 2. The extract was dissolved in distilled water and was freshly prepared every day before administration. Rats in the control group were given 10 mL/kg of distilled water daily by gavage. Conventional diets and water were freely available to all rats during the experiment.

#### 2.4.2. Animal Observation

Body weight of the rats was recorded on the first day of administration, once a week thereafter and on the day of necropsy. Food consumption was recorded twice a week throughout the experiment. During each recording, the amount of food consumed by each rat was determined by subtracting the weight of food left from the weight of food given and then dividing it by the number of rats in the cage. Weekly and total food utilization rates were calculated. Signs of toxicity and mortality were also monitored daily throughout the experiment.

#### 2.4.3. Hematology and Serum Biochemical Monitoring

On Day 45, blood samples were collected from the ophthalmic veins of rats and used for hematological examination. Blood samples were mixed in ethylenediaminetetraacetic acid (EDTA) anticoagulant and analyzed using a Sysmex XT-1800 automated hematological analyzer (Sysmex, Kobe, Japan). The following hematological parameters were examined: white blood cell count (WBC), red blood cell count (RBC), hemoglobin concentration (HGB), hematocrit (HCT), platelet count (PLT), mean platelet volume (MPV), mean corpuscular volume (MCV), mean corpuscular hemoglobin (MCH), mean corpuscular hemoglobin concentration (MCHC), number and percentage of neutrophils (NEUT), number and percentage of lymphocytes (LYM), number and percentage of monocytes (MONO), number and percentage of eosinophils (EO), and number and percentage of basophils (BASO). Reagents for the hematology were purchased from Sysmex (Kobe, Japan).

At the end of the experiment (Day 90), rats were fasted overnight with free access to sterilized tap water. At the end of exposure, animals were anaesthetized with pentobarbital and sacrificed. Blood samples were collected from the arteria abdominalis of rats. Hematological examination was conducted as described above. For biochemical examination, blood samples were centrifuged (2,500 rpm) for 10 min, and serum was collected and analyzed for alanine transaminase (ALT), aspartate transaminase (AST), total cholesterol (TC), triglyceride (TG), total protein (TP), albumin (ALB), glucose (GLU), blood urea nitrogen (BUN), and creatinine (CR) using an Olympus AU400 analyzer (Olympus, Tokyo, Japan). All the commercial kits used for biochemical examinations were purchased from Beijing Wantai BioPharm Company (Beijing, China) [27].

#### 2.4.4. Organ Weights and Histopathology

A gross anatomical observation was conducted on each animal after the blood collection. Absolute weights of the liver, spleen, kidneys, and testes were measured, and the ratios of organ weight to body weight were calculated. Specimens of major organs and tissues, including the liver, spleen, kidneys, testes, stomach, duodenum, ovaries, brain, heart, and lung, were examined for potential histopathological changes. Specimens were fixed in 10% neutral buffered formaldehyde for more than 48 h, dehydrated (Leica ASP300 S), and processed into paraffin blocks (Leica EG1150H). Sections (3 *μ*m) of organs and tissues were sliced and stained with hematoxylin-eosin (Leica ST5020 and Leica CV5030). Sections were examined under a Leica DM 6000B microscope (Wetzler, Germany). For each animal, one slide of each organ or tissue was examined with 100-fold magnification. Digital images were taken when histopathological changes were observed. Histopathological changes were scored based on severity of tissue/cellular damage into four levels: normal (−), mild (+), moderate (++), and severe (+++). Two different evaluations were performed following the histopathological examination: the comparison of the number of rats with histopathological changes and scores of damages between treated and control groups.

### 2.5. Statistical Analysis

Data were presented as the mean ± standard deviation of 10 rats of the same sex in each group, unless otherwise noted. Data of the subchronic toxicity study were analyzed by the chi-square test and one-way analysis of variance (ANOVA) followed by Dunnett's test using SPSS version 16.0 (SPSS Inc, Chicago, Illinois, USA). A *p* value < 0.05 was considered statistically significant. Figures were drawn with GraphPad PRISM software (GraphPad Software, San Diego, California, USA).

## 3. Results

### 3.1. Polypeptide Pattern of the Extract

**Figure 1:**
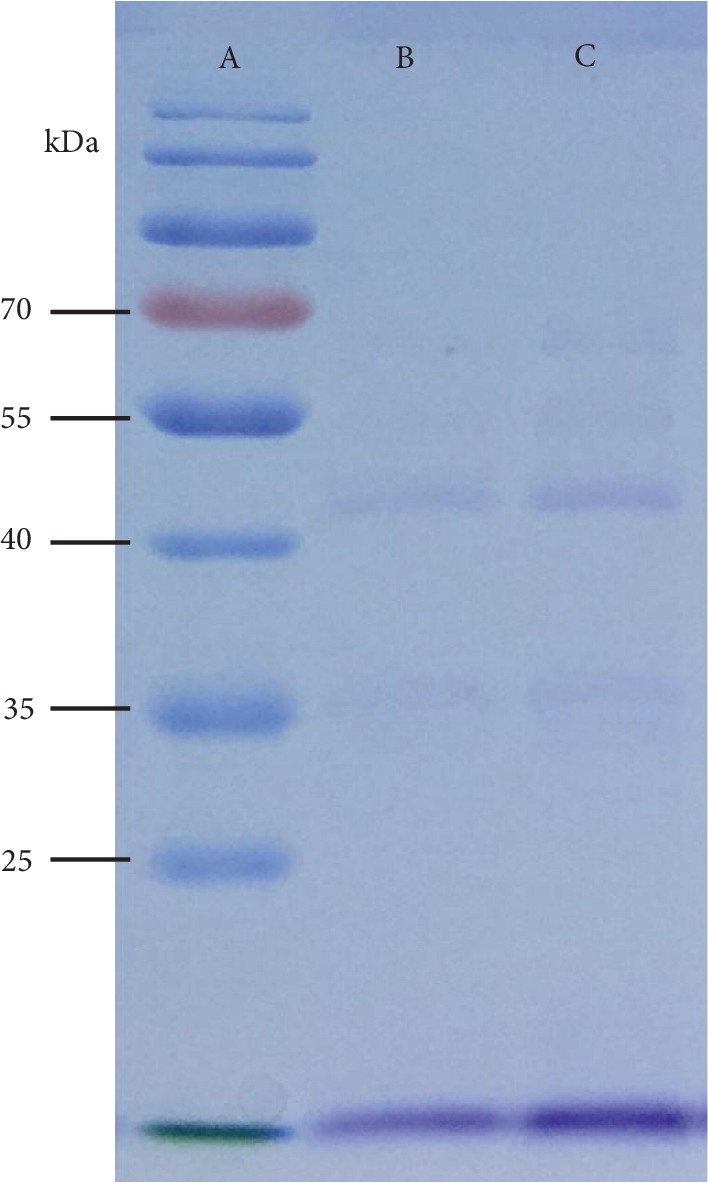
Polypeptide pattern of the WKB extract under SDS-PAGE separation. Lane A, molecular weight of markers; lane B, WKB extract; lane C, commercial WKB extract (Phase 2®).

Polypeptide pattern of the WKB extract under SDS-PAGE separation is shown in Figure 1. The WKB extract had a similar polypeptide pattern as the commercial product Phase 2®, with the highest match peptide fractions around 36 and 45 kDa.

### 3.2. Subchronic Oral Toxicity Study in Rats

**Figure 2:**
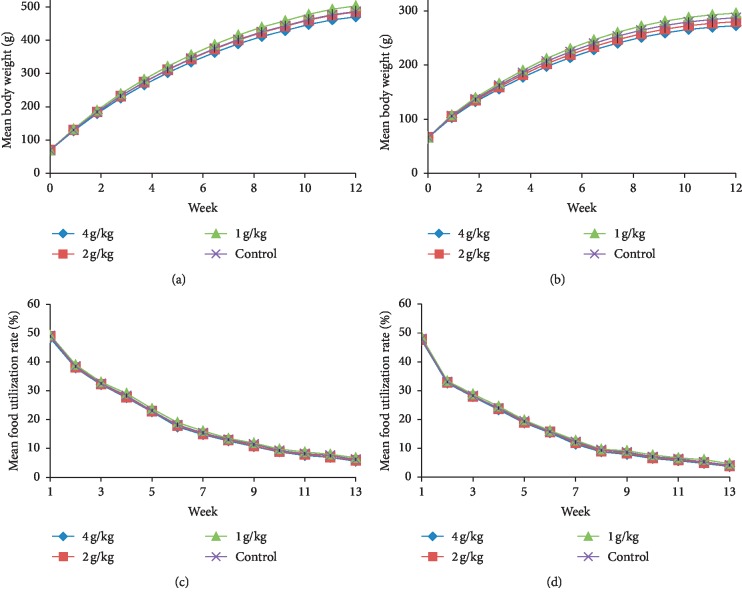
Average weekly body weights of (a) male and (b) female SD rats and food utilization rates of (c) male and (d) female SD rats treated with white kidney bean extract for 90 days (represented by mean, *n* = 10).

During the 90-day subchronic oral toxicity study, no apparent adverse effects or mortalities were observed in rats treated with WKB extract. Moreover, body weight gains and food consumption were unaffected by the treatment (Figure 2 and Table 1). There was no statistically significant difference in body weight and weight gain between treated and untreated rats of both sexes (*p* > 0.05). Average food utilization rates were 18.1 and 15.9% for the male and female rats in the control group, respectively. Treated rats did not demonstrate any dose-related changes in food utilization rates, total food consumption, and average food utilization rates as compared to control rats (*p* > 0.05).

The hematological and serum biochemical parameters of rats treated with WKB extract were shown in Tables 2–4. As compared to control, WKB extract did not induce any significant changes in biochemical and hematological parameters in rats on both Day 45 and Day 90.

**Figure 3:**
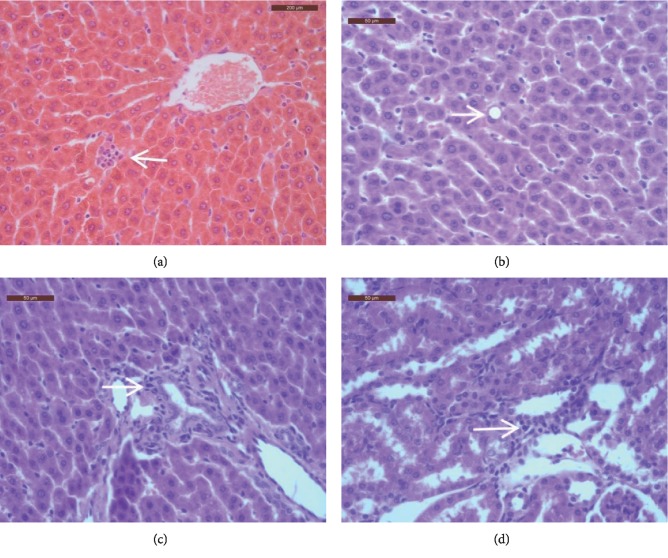
Representative histopathological changes in the liver and kidneys of rats treated with 4 g/kg/day white kidney bean extract for 90 days: (a) spotty necrosis of hepatocytes in the hepatic lobules; (b) fatty degeneration of hepatocytes in the hepatic lobules; (c) inflammatory cell infiltration in the portal tract; (d) cells infiltration in renal interstitium. Arrows indicate histopathological changes. The scale bars are 200 *μ*m for (a) and 50 *μ*m for (b–d).

Absolute and relative organ weights of the liver, spleen, kidney, and testes in rats from treatment groups were similar to those of the control group (*p* > 0.05, Table 5). Gross necropsy revealed no signs of pathological lesions in both treated as well as untreated rats. Therefore, histopathological examinations were conducted only on rats in the 4 g/kg (the highest dose) treatment and the control group. Several types of minor histopathological changes were observed in rats from both the treatment and the control group, including inflammatory cell infiltration in portal duct areas of the liver, mild spotty necrosis of hepatocytes in the hepatic lobules, mild fatty degeneration of hepatocytes in the hepatic lobules, and cell infiltration in renal interstitium (Table 6 and Figure 3). Nonetheless, histopathological changes in the treated group were considered as spontaneous because the lesions were comparable to those observed in the control group. In addition, no histopathological changes were evident in the brain, heart, spleen, thyroid gland, pancreatic gland, adrenal gland, mesenteric lymph nodes, small intestine, jejunum, ileum, prostate, bladder, testes, and ovaries of rats from these two groups.

## 4. Discussion

Common beans contain high levels of nutrients and natural products that are associated with antioxidant, antihyperglycemic, and anticancer effects [2]. This legume is a good source of protein, starch, and dietary fiber, and it accounts for approximately 50% of global grain legume consumption in humans [5]. Common beans are suitable for application in a wide range of dietary supplements because of the presence of abundant nutritional substances and bioactive phytochemicals. Among them, *α*-AI extracted from common bean has attracted significant attention. Common beans contain 3 isoforms of *α*-AI: *α*-AI1, *α*-AI2, and *α*-AIL [11]. The most active isoform, *α*-AI1, was reported to be a glycoprotein with an N-glycosylation site and a molecular weight of 36 to 55 kDa [16, 28]. These lectin-like inhibitors could noncovalently bind to *α*-amylase, resulting in blocking of the active site of the *α*-amylase [29]. In this study, the polypeptide pattern of WKB extract was similar to that of the commercial product Phase 2®, with two highest match peptide fractions around 36 and 45 kDa, which corresponded to the *α*-AI1 isoform. WKBs of different origins and accessions have been reported to have relatively high *α*-amylase inhibitory activity, ranging from 1,840 to 5,777 U/g [5]. The *α*-amylase inhibitory activity of the WKB extract used in the present study (2,157 U/g) was within the range of the reported values.

Safety evaluation of several marketed dietary supplements containing the WKB extract as an ingredient has been reported in literature. In the preliminary assessment of Phase 2®, a standardized extract of the WKB with *α*-amylase inhibitory activity, doses of 5 g/kg given acutely and 1 g/kg given subchronically for 90 days did not produce any significant toxic effects in Wistar rats [21]. In a 28-day oral study, the no-observed-adverse-effect-level (NOAEL) of Phase 2® was determined to be 2.5 g/kg/day for both male and female rats [20]. Toxicity studies with Blockal, a dietary supplement containing Phase 2®, showed that the acute 14-day oral LD_50_ was >3 g/kg bw (i.e., 1668 mg/kg of Phase 2®) and the 28-day NOAEL was 2 g/kg bw/day (i.e., 1112 mg/kg of Phase 2®) [19]. Another 28-day study in rats indicated that the NOAEL of steady-fiber granule, a functional food mixture containing 5% WKB extract, was 5 g/kg/day [30]. A recent study examined the toxicity of *α*-AI extracted from the WKB in a 21-day oral toxicity study in Wistar rats [16]. The results showed that although food intake was influenced by 8.1 mg/kg *α*-AI extract, body weight of rats treated with the extract did not differ significantly from the control. However, changes of weight and length in the digestive tract were observed [16].

In the present study, the subchronic oral toxicity study was used to assess the toxicity of the WKB extract. The results of the repeated-dose 90-day oral toxicity study produced no treatment-related changes in body weight, absolute organ weight, relative organ weight, food consumption, blood chemistry, and hematology in male and female SD rats. Since histopathological changes in the liver, stomach, and kidneys in high-dose (4 g/kg)-treated rats were minor and similar to those observed in the control animals, they were considered as spontaneous lesions. Moreover, no apparent histopathological changes were detected in other major organs or tissues of treated rats. Based on the findings of this study, the NOAEL of the WKB extract was determined to be 4 g/kg/day for SD rats, by oral gavage for 90 days. Using a nominal 100-fold safety factor [31], the safe dose of WKB extract for human can be determined to be 40 mg/kg/day (i.e., 2.8 g/day for a 70 kg person).

WKB extracts containing *α*-AIs have been shown to possess antiobesity, antidiabetic, and glycaemic control effects [9, 10, 13]. However, in the present study, only minor effects on blood glucose and obesity-related parameters (e.g., body weight, cholesterol, and triglyceride) were observed in rats treated with WKB extract. One possible explanation of this discrepancy is that the healthy rats are probably more resistant to effects of *α*-AI due to high amylase activity as compared to obese rats. This explanation is supported by several previous studies which demonstrated a lower pancreatic amylase activity in obese individuals as compared to lean individuals in both rats and humans [32, 33].

Under the experimental conditions of the current study, the WKB extract might not have been able to inhibit the *α*-amylase activity of healthy rats. Consequently, no abnormal glucose metabolism or weight loss was found. This result is in agreement with a previous study, which used Phase 2® as a test substance. Phase 2® (>3,000 U/g *α*-amylase inhibiting activity) at doses up to 2,500 mg/kg did not significantly affect body weight or nutritional effects of rats treated subchronically [20]. The NOAEL of Phase 2® was determined to be 2.5 g/kg/day, and the daily intake dose of Phase 2® was recommended to be 85 mg/kg/day (6 g for a 70 kg person) from overall food and dietary supplement, when a 30-fold safety factor was applied [20]. Another report suggested the maximum oral intake of Phase 2® to be 10 g/day for a 70 kg person [34]. Taken together, the maximum daily oral intake of WKB extract up to 2.8 g/day for a 70 kg person is expected to be safe in humans.

## 5. Conclusion

This study evaluated the potential toxicity of a WKB extract in SD rats using a 90 d repeated-dose subchronic toxicity study. The results of the current study showed that the administration of WKB extract to rats at doses up to 4 g/kg bw did not induce significant effect in the body and organ weight, food consumption, hematology, serum biochemistry, and histopathology. Under the experimental conditions of this study, the NOAEL was determined to be >4 g/kg bw for male and female SD rats, by oral gavage for 90 days. Further study is needed to evaluate the efficacy of the extract on *α*-amylase.

## Figures and Tables

**Table 1:** Total body weight gain, food consumption, and food utilization rate of rats treated with white kidney beans for 90 days.

Dose (g/kg)	Total body weight gain (g)	Total food consumption (g)	Average food utilization rate (%)
Male			
4	400.3 ± 42.2	2305.9 ± 166.9	17.4 ± 0.9
2	414.6 ± 34.1	2366.1 ± 125.8	17.5 ± 0.7
1	400.3 ± 42.2	2289.8 ± 249.6	18.9 ± 0.7
Control	416.2 ± 46.1	2293.1 ± 215.1	18.1 ± 0.8

Female			
4	207.0 ± 21.0	1371.2 ± 95.3	15.1 ± 0.5
2	213.8 ± 25.3	1383.9 ± 110.9	15.4 ± 0.7
1	229.9 ± 44.4	1361.3 ± 199.2	16.8 ± 0.9
Control	221.4 ± 26.3	1386.5 ± 110.7	15.9 ± 1.0

Note: values represent mean ± standard deviation (*n* = 10). Average food utilization rate (%) = (total body weight gain/total food consumption) × 100%. Values of the treatment groups did not differ statistically from the control according to one-way ANOVA at *p* < 0.05.

**Table 2:** Hematology examination of rats treated with white kidney beans on Day 45.

Dose (g/kg)	HGB (g/L)	RBC (10^12^/L)	PLT (10^9^/L)	WBC (10^9^/L)	LYM (%)	NEUT (%)	MONO (%)	EO (%)	BASO (%)
Male									
4	153.2 ± 4.4	8.34 ± 0.37	907.6 ± 104.9	7.47 ± 0.97	77.2 ± 3.5	11.4 ± 1.6	10.38 ± 4.32	0.95 ± 0.33	0.09 ± 0.10
2	153.6 ± 3.4	8.78 ± 0.76	859.6 ± 83.7	7.56 ± 1.14	75.5 ± 3.3	10.9 ± 3.4	12.88 ± 4.15	0.66 ± 0.42	0.10 ± 0.11
1	153.4 ± 5.1	8.48 ± 0.58	853.7 ± 65.2	7.91 ± 0.96	76.8 ± 4.1	11.7 ± 4.8	10.70 ± 5.47	0.82 ± 0.51	0.07 ± 0.08
Control	152.8 ± 5.0	8.60 ± 0.47	877.8 ± 107.1	7.73 ± 1.08	78.5 ± 2.1	10.4 ± 2.2	10.15 ± 3.05	0.85 ± 0.41	0.08 ± 0.09

Female									
4	155.5 ± 3.3	8.46 ± 0.43	854.8 ± 104.5	7.84 ± 0.82	77.6 ± 4.4	11.7 ± 3.4	9.58 ± 3.92	0.98 ± 0.44	0.10 ± 0.09
2	154.2 ± 5.6	8.60 ± 0.51	826.3 ± 127.8	7.95 ± 1.14	76.5 ± 3.8	10.7 ± 5.4	11.96 ± 8.26	0.74 ± 0.55	0.08 ± 0.09
1	151.8 ± 3.9	8.35 ± 0.38	826.9 ± 79.1	7.40 ± 1.21	77.8 ± 2.9	10.8 ± 3.4	10.33 ± 3.80	0.94 ± 0.73	0.07 ± 0.08
Control	153.9 ± 6.0	8.61 ± 0.53	833.1 ± 73.4	7.73 ± 0.80	78.4 ± 5.4	11.7 ± 3.0	8.85 ± 4.81	0.95 ± 0.32	0.08 ± 0.09

Note: values represent mean ± standard deviation (*n* = 10). HGB, hemoglobin concentration; RBC, red blood cell count; PLT, platelet count; WBC, white blood cell count; LYM, percent of lymphocytes; NEUT, percent of neutrophils; MONO, percent of monocytes; EO, percent of eosinophils; BASO, percent of basophils. Values of the treatment groups did not differ statistically from the control according to one-way ANOVA at *p* < 0.05.

**Table 3:** Hematology examination of rats treated with white kidney beans on Day 90.

Dose (g/kg)	HGB (g/L)	RBC (10^12^/L)	PLT (10^9^/L)	WBC (10^9^/L)	LYM (%)	NEUT (%)	MONO (%)	EO (%)	BASO (%)
Male									
4	156.0 ± 5.8	8.91 ± 0.53	891.2 ± 74.0	7.37 ± 1.29	80.3 ± 3.5	11.1 ± 3.5	7.34 ± 2.35	1.21 ± 1.13	0.07 ± 0.11
2	156.2 ± 7.5	9.03 ± 0.53	869.4 ± 118.8	7.83 ± 1.53	78.4 ± 1.6	13.3 ± 1.8	7.28 ± 1.24	0.93 ± 0.88	0.08 ± 0.11
1	154.6 ± 6.0	8.04 ± 0.51	880.2 ± 70.3	6.94 ± 0.85	78.7 ± 2.6	13.0 ± 2.2	7.02 ± 1.50	1.24 ± 0.51	0.10 ± 0.12
Control	155.2 ± 4.7	8.56 ± 0.72	868.0 ± 101.0	6.80 ± 1.14	79.2 ± 2.3	11.8 ± 2.2	7.61 ± 1.69	1.27 ± 1.16	0.09 ± 0.10

Female									
4	157.0 ± 7.7	8.68 ± 0.46	884.4 ± 133.6	6.72 ± 1.20	79.2 ± 2.1	13.0 ± 2.0	6.70 ± 1.50	1.06 ± 0.64	0.10 ± 0.09
2	154.0 ± 5.2	8.36 ± 0.85	863.4 ± 79.9	7.47 ± 0.87	79.5 ± 2.1	13.2 ± 1.8	6.26 ± 1.83	1.01 ± 0.71	0.08 ± 0.09
1	152.2 ± 7.0	8.07 ± 0.57	866.4 ± 102.4	6.68 ± 1.60	78.4 ± 2.1	13.5 ± 2.1	7.02 ± 1.86	1.10 ± 0.68	0.07 ± 0.11
Control	158.0 ± 4.3	8.52 ± 0.39	886.0 ± 117.5	7.03 ± 1.39	78.4 ± 4.1	13.4 ± 4.0	6.92 ± 1.85	1.13 ± 0.49	0.09 ± 0.07

Note: values represent mean ± standard deviation. HGB, hemoglobin concentration; RBC, red blood cell count; PLT, platelet count; WBC, white blood cell count; LYM, percent of lymphocytes; NEUT, percent of neutrophils; MONO, percent of monocytes; EO, percent of eosinophils; BASO, percent of basophils. Values of the treatment groups did not differ statistically from the control according to one-way ANOVA at *p* < 0.05.

**Table 4:** Serum biochemical examination of rats treated with white kidney beans on day 90.

Dose (g/kg)	ALT (U/L)	AST (U/L)	TC (mmol/L)	TG (mmol/L)	TP (g/L)	ALB (g/L)	GLU (mmol/L)	BUN (mmol/L)	Cr (*μ*mol/L)
Male									
4	63.10 ± 18.63	104.51 ± 11.16	2.10 ± 0.26	1.03 ± 0.31	71.69 ± 4.96	35.83 ± 3.25	5.23 ± 0.53	7.21 ± 0.86	53.03 ± 4.65
2	64.14 ± 13.16	107.46 ± 6.80	2.09 ± 0.26	1.01 ± 0.21	71.82 ± 3.58	36.33 ± 1.82	5.34 ± 0.62	6.82 ± 0.77	54.26 ± 3.74
1	53.54 ± 16.30	99.78 ± 10.90	1.98 ± 0.36	1.39 ± 0.45	68.07 ± 4.58	37.14 ± 2.42	5.45 ± 0.53	7.37 ± 1.35	56.39 ± 6.26
Control	58.18 ± 15.35	100.76 ± 8.42	2.07 ± 0.42	1.02 ± 0.41	69.75 ± 6.72	37.28 ± 2.83	5.85 ± 1.01	6.69 ± 1.11	53.78 ± 3.56
Female									
4	58.97 ± 18.14	109.34 ± 8.17	2.09 ± 0.24	1.12 ± 0.25	71.95 ± 5.49	35.75 ± 2.76	5.15 ± 0.65	7.24 ± 1.06	56.69 ± 3.89
2	52.80 ± 8.68	104.66 ± 5.66	2.14 ± 0.43	1.02 ± 0.23	72.47 ± 4.97	36.58 ± 2.56	5.75 ± 0.76	6.71 ± 1.12	54.16 ± 3.87
1	42.99 ± 13.78	101.81 ± 10.00	2.10 ± 0.16	1.17 ± 0.30	71.60 ± 3.33	37.43 ± 3.18	5.22 ± 0.69	6.94 ± 1.21	58.09 ± 9.95
Control	46.43 ± 10.56	102.20 ± 7.76	2.07 ± 0.13	1.09 ± 0.21	71.26 ± 3.39	37.76 ± 4.32	5.35 ± 0.40	6.90 ± 1.39	54.51 ± 5.29

Note: values represent mean ± standard deviation (*n* = 10). ALT, alanine transaminase; AST, aspartate transaminase; TC, total cholesterol; TG, triglyceride; TP, total protein; ALB, albumin; GLU, glucose; BUN, blood urea nitrogen; CR, creatinine. Values of the treatment groups did not differ statistically from the control according to one-way ANOVA at *p* < 0.05.

**Table 5:** Absolute and relative organ weights of rats treated with white kidney beans for 90 days.

Dose (g/kg)	Fasting body weight (g)	Absolute organ weight (g)				Relative organ weight (%)			
		Liver	Kidneys	Spleen	Testes	Liver	Kidneys	Spleen	Testes
Male									
4	456.5 ± 41.8	9.126 ± 1.093	2.420 ± 0.314	0.726 ± 0.099	2.963 ± 0.283	2.027 ± 0.383	0.537 ± 0.105	0.161 ± 0.032	0.657 ± 0.106
2	471.1 ± 30.5	9.558 ± 0.857	2.545 ± 0.220	0.745 ± 0.087	3.161 ± 0.285	2.032 ± 0.167	0.542 ± 0.059	0.158 ± 0.018	0.675 ± 0.091
1	489.3 ± 59.2	9.340 ± 0.540	2.438 ± 0.149	0.725 ± 0.098	3.027 ± 0.192	1.937 ± 0.282	0.504 ± 0.055	0.151 ± 0.033	0.625 ± 0.065
Control	473.1 ± 46.2	9.436 ± 0.710	2.460 ± 0.116	0.737 ± 0.084	3.155 ± 0.204	2.011 ± 0.237	0.524 ± 0.057	0.157 ± 0.025	0.674 ± 0.085
Female									
4	262.8 ± 22.1	6.647 ± 0.418	1.672 ± 0.067	0.530 ± 0.077	NA	2.548 ± 0.283	0.640 ± 0.049	0.203 ± 0.030	NA
2	270.8 ± 29.0	6.702 ± 0.364	1.682 ± 0.079	0.525 ± 0.071	NA	2.500 ± 0.290	0.628 ± 0.075	0.196 ± 0.031	NA
1	286.2 ± 46.0	6.526 ± 0.582	1.708 ± 0.102	0.512 ± 0.042	NA	2.338 ± 0.433	0.608 ± 0.078	0.183 ± 0.033	NA
Control	277.8 ± 24.8	6.515 ± 0.534	1.709 ± 0.118	0.514 ± 0.041	NA	2.371 ± 0.361	0.621 ± 0.083	0.187 ± 0.026	NA

Note: values represent mean ± standard deviation (*n* = 10). Relative organ weight (%) = (absolute organ weight/fasting body weight) × 100%. NA, not available. Values of the treatment groups did not differ statistically from the control according to one-way ANOVA at *p* < 0.05.

**Table 6:** Histopathology examination of rats treated with white kidney beans for 90 days.

Organ (histopathology changes)	Male		Female	
	4 g/kg	Control	4 g/kg	Control
Liver (inflammatory cell infiltration in portal duct areas)	0	1	2	1
Liver (mild spotty necrosis of hepatocytes)	0	1	1	1
Liver (mild fatty degeneration of hepatocytes)	1	1	0	0
Kidney (cell infiltration in renal interstitium)	1	1	1	1

Note: values represent numbers of rats with histopathology changes in 10 rats of each group.

## Data Availability

The data used to support the findings of this study are included within the article.

## Abbreviation

*α*-AI: Alpha-amylase inhibitors
WKBs: White kidney beans
SPF: Specific pathogen free
SD: Sprague-dawley
MTD: Maximum tolerated dose
bw: Body weight
EDTA: Ethylene diamine tetraacetic acid
HGB: Hemoglobin concentration
RBC: Red blood cell count
PLT: Platelet count
WBC: White blood cell count
LYM: Percent of lymphocytes
NEUT: Percent of neutrophils
MONO: Percent of monocytes
EO: Percent of eosinophils
BASO: Percent of basophils
AST: Aspartate transaminase
ALT: Alanine transaminase
BUN: Blood urea nitrogen
CR: Creatinine
TC: Total cholesterol
TG: Triglyceride
TP: Total protein
ALB: Albumin
GLU: Glucose
ANOVA: One-way analysis of variance
NOAEL: No-observed-adverse-effect level.
